# A phase I and pharmacokinetic study of the combination of capecitabine and docetaxel in patients with advanced solid tumours

**DOI:** 10.1054/bjoc.2000.1160

**Published:** 2000-06-02

**Authors:** L C Pronk, P Vasey, A Sparreboom, B Reigner, A S Th Planting, R J Gordon, B Osterwalder, J Verweij, C Twelves

**Affiliations:** Rotterdam Cancer Institute (Daniel den Hoed Kliniek) and University Hospital, Rotterdam, The Netherlands; Cancer Research Campaign, Department of Medical Oncology, Beatson Oncology Centre, Glasgow, UK; Quintiles Oncology, Strasbourg, France; Hoffmann-La Roche, Basel, Switzerland

**Keywords:** phase I, pharmacokinetics, capecitabine, docetaxel

## Abstract

Capecitabine and docetaxel are both active against a variety of solid tumours, while their toxicity profiles only partly overlap. This phase I study was performed to determine the maximum tolerated dose (MTD) and side-effects of the combination, and to establish whether there is any pharmacokinetic interaction between the two compounds. Thirty-three patients were treated with capecitabine administered orally twice daily on days 1–14, and docetaxel given as a 1 h intravenous infusion on day 1. Treatment was repeated every 3 weeks. The dose of capecitabine ranged from 825 to 1250 mg m^–2^twice a day and of docetaxel from 75 to 100 mg m^–2^. The dose-limiting toxicity (DLT) was asthenia grade 2–3 at a dose of 1000 mg m^–2^bid of capecitabine combined with docetaxel 100 mg m^–2^. Neutropenia grade 3–4 was common (68% of courses), but complicated by fever in only 2.4% of courses. Other non-haematological toxicities were mild to moderate. There was no pharmacokinetic interaction between the two drugs. Tumour responses included two complete responses and three partial responses. Capecitabine 825 mg m^–2^twice a day plus docetaxel 100 mg m^–2^was tolerable, as was capecitabine 1250 mg m^–2^twice a day plus docetaxel 75 mg m^–2^. © 2000 Cancer Research Campaign


British Journal of Cancer (2000) **83**(1), 22–29
© 2000 Cancer Research Campaign
doi: 10.1054/ bjoc.2000.1160, available online at http://www.idealibrary.com on IDEAL®

### A phase I and pharmacokinetic study of the combination of capecitabine and docetaxel in patients with advanced solid tumours

**LC Pronk[1], P Vasey[2], A Sparreboom[1], B Reigner[3], ASTh Planting[1], RJ Gordon[3], B Osterwalder[3], J Verweij[1] and C Twelves[2]**

[1] Rotterdam Cancer Institute (Daniel den Hoed Kliniek) and University Hospital, Rotterdam, The Netherlands; [2] Cancer Research Campaign, Department of Medical Oncology, Beatson Oncology Centre, Glasgow, UK; [3]Quintiles Oncology, Strasbourg, France; Hoffmann-La Roche, Basel, Switzerland

**Summary** Capecitabine and docetaxel are both active against a variety of solid tumours, while their toxicity profiles only partly overlap. This phase I study was performed to determine the maximum tolerated dose (MTD) and side-effects of the combination, and to establish whether there is any pharmacokinetic interaction between the two compounds. Thirty-three patients were treated with capecitabine administered orally twice daily on days 1–14, and docetaxel given as a 1 h intravenous infusion on day 1. Treatment was repeated every 3 weeks. The dose of capecitabine ranged from 825 to 1250 mg $m^{-2}$ twice a day and of docetaxel from 75 to 100 mg $m^{-2}$. The dose-limiting toxicity (DLT) was asthenia grade 2–3 at a dose of 1000 mg $m^{-2}$ bid of capecitabine combined with docetaxel 100 mg $m^{-2}$. Neutropenia grade 3–4 was common (68% of courses), but complicated by fever in only 2.4% of courses. Other non-haematological toxicities were mild to moderate. There was no pharmacokinetic interaction between the two drugs. Tumour responses included two complete responses and three partial responses. Capecitabine 825 mg $m^{-2}$ twice a day plus docetaxel 100 mg $m^{-2}$ was tolerable, as was capecitabine 1250 mg $m^{-2}$ twice a day plus docetaxel 75 mg $m^{-2}$. © 2000 Cancer Research Campaign

**Keywords**: phase I; pharmacokinetics; capecitabine; docetaxel

Capecitabine (Xeloda™, Hoffmann-La Roche, Basel, Switzerland) is an orally administered prodrug of 5-fluorouracil (5-FU) that passes intact through the intestinal mucosa. It is activated by a cascade of three enzymes to 5′-deoxy-5-fluorocytidine (5′-DFCR), then to 5′-deoxy-5-fluorouridine (5′-DFUR), resulting in an intratumoural release of 5-FU. This final, tumour-selective, enzyme reaction is mediated by the tumour-associated angiogenic factor, thymidine phosphorylase (TP). Capecitabine is cytotoxic only after conversion to 5′-DFUR and 5-FU. In human cancer xenograft murine models, capecitabine was substantially more active than 5-FU against colon CXF 280 and HCT 116, gastric MKN 45 and GXF 97, breast MAXF 401 and MX-1, cervical YUMOTO, HT-3 and SK-OV-3, ovarian NAKAJIMA, bladder SCABER and hepatoma IH-3. This anti-tumour activity in mice correlated with tumour 5-FU and blood 5′-DFUR levels (Investigational drug brochure: capecitabine 1997). The cytotoxicity of capecitabine correlated well with the activity ratio in tumours of TP and dihydropyrimidine dehydrogenase, the enzymes for the conversion of capecitabine to 5-FU and the catabolism of 5-FU respectively (Ishikawa et al, 1997). Furthermore, in preclinical studies, paclitaxel and docetaxel were more active in combination with capecitabine than with 5-FU or UFT. Recently, it was demonstrated that thymidine phosphorylase is up-regulated in murine model systems exposed to taxanes (Sawada et al, 1998).

*Received 20 October 1999*
*Revised 1 February 2000*
*Accepted 17 February 2000*

*Correspondence to:* LC Pronk, Hospital Universitario ‘12 de Octubre’, Servicio de Oncología Médica, Carretera de Andalucia Km 5,4, 28041 Madrid, Spain

In phase I studies of capecitabine as a single agent different treatment schedules were investigated. Capecitabine was given either continuously for 6 weeks or using an intermittent twice daily schedule (Taguchi et al, 1996; Twelves et al, 1996; Budman et al, 1998; Mackean et al, 1998). Each of those schedules were active and common adverse events included diarrhoea, hand–foot syndrome, nausea, vomiting, stomatitis and asthenia.

In a randomized phase II study of capecitabine in patients with advanced colorectal cancer the following three treatment schedules were evaluated: capecitabine 1331 mg $m^{-2}$ $day^{-1}$ continuously, capecitabine 2510 mg $m^{-2}$ $day^{-1}$ for 14 days repeated every 21 days and capecitabine 1657 mg $m^{-2}$ $day^{-1}$ combined with leucovorin 60 mg $day^{-1}$ orally (p.o.) given intermittently (Findlay et al, 1997). Time to progression reported for these three administration schedules was 17, 30 and 24 weeks respectively. Furthermore, dose intensity appeared highest with the intermittent single-agent schedule which was therefore selected for phase III evaluation. Capecitabine was recently registered in the USA for treatment of patients with breast cancer refractory to paclitaxel and anthracyclines (Blum et al, 1999).

Docetaxel (Taxotere™, Rhône-Poulenc Rorer, Antony, France) is an antimicrotubule agent that enhances polymerization of tubulin into stable microtubules and inhibits microtubule depolymerization. This disrupts the equilibrium within the microtubule system and ultimately leads to cell death (Guerrite-Voegelein et al, 1991; Ringel and Horwitz, 1991; Rowinsky and Donehower, 1991). In phase I studies of single-agent docetaxel the major dose-limiting toxicity (DLT) was a short-lasting, dose-dependent, schedule independent and non-cumulative neutropenia (Aapro et al, 1992; Pazdur et al, 1992; Bisset et al, 1993; Burris et al, 1993; Extra et al, 1993; Tomiak et al, 1993). Based on these phase I

studies the recommended single-agent dose and schedule for docetaxel was 100 mg $m^{-2}$ given as a 1-h infusion every 3 weeks.

Phase II studies on docetaxel among others showed activity in breast cancer (Ten Bokkel-Huinink et al, 1994; Chevallier et al, 1995), non-small-cell lung cancer (Cerny et al, 1994; Fossella et al, 1995; Miller et al, 1995), head and neck cancer (Catimel et al, 1994), gastric cancer (Sulkes et al, 1994), melanoma (Aamdal et al, 1994), soft tissue sarcoma (Van Hoesel et al, 1994) and pancreatic cancer (De Forni et al, 1994). Again, the most important side-effect was early but short-lasting neutropenia, that was complicated by infection in 20% of the patients (Pronk et al, 1995). Alopecia was common, but other toxicities were usually mild and included nausea, vomiting, diarrhoea, mucositis, asthenia, infrequent hypersensitivity reactions, skin reactions, nail changes, mild sensory neuropathy and fluid retention. Corticosteroid premedication has markedly reduced the incidence of hypersensitivity reactions (Schrijvers et al, 1993) as well as the severity of fluid retention (Piccart et al, 1997), and is now standard therapy.

In this phase I study the combination of capecitabine with docetaxel was studied because given as single agents both drugs are active in a variety of cancers and their toxicity profiles are only partly overlapping. The aims of this study were: to determine the maximum tolerated dose (MTD); to determine the safety profile of the combination; to evaluate if there is any pharmacokinetic interaction between capecitabine and its metabolites and docetaxel; to report any evidence of anti-tumour activity.

### PATIENTS AND METHODS

### Eligibility

Patients with histologically confirmed solid tumours for whom no other therapy with greater potential benefit existed than the combination of capecitabine with docetaxel, were entered into this study. The study was approved by the institutional ethics committee.

Eligibility criteria included: age 18 years and older; Karnofsky performance status ≥ 70; no more than two prior single-agent chemotherapy regimens or one prior combination chemotherapy regimen; no prior treatment with docetaxel and/or capecitabine; normal bone marrow (haemoglobin > 9.0 g $dl^{-1}$, granulocytes > $1.5 \times 10^9$ $l^{-1}$, platelet count > $100 \times 10^9$ $l^{-}$), renal (serum creatinine < 1.5 × upper normal limit) and hepatic function (bilirubin < 1.25 × upper normal limit, alkaline phosphatase < 2.5 × upper normal limit, and transaminases < 1.5 × upper normal limit); uric acid < 1.25 × upper normal limit, calcium < 2.88 mmol $l^{-1}$, no clinically significant cardiac disease or myocardial infarction within the last 12 months; no radiation therapy within 4 weeks of treatment start; no major surgery to the gastrointestinal tract, the liver or kidney within 4 weeks of study entry which may impact on the pharmacokinetics of capecitabine or docetaxel; no participation in any investigational drug study within 4 weeks preceding treatment start; no history of peptic ulcer, ulcerative colitis, ulcerative stomatitis and/or lack of physical integrity of the upper intestinal tract. All patients provided written informed consent.

### Pretreatment and follow-up studies

Before the start of treatment a medical history was taken and physical examination, laboratory studies, electrocardiogram, chest X-ray and imaging studies, if appropriate, were performed. Laboratory studies included a full blood count with differential white count, sodium, potassium, creatinine, uric acid, serum calcium, total protein, albumin, total bilirubin, ALAT, ASAT, alkaline phosphatase and urinalysis.

History, physical examination and toxicity scoring according to National Cancer Institute Common Toxicity Criteria (NCI-CTC) (Brundage et al, 1993) were performed every 3 weeks and laboratory studies weekly. Formal tumour assessments were performed after every 2 courses of chemotherapy according to standard World Health Organization (WHO) response criteria (WHO Handbook 1979).

### Drug administration

Patients received treatment every 3 weeks. Docetaxel was administered on day 1 of each cycle as a 1-h intravenous (i.v.) infusion. Capecitabine was to be administered orally within 30 min after the end of a meal. The first cycle of capecitabine was given twice daily starting on days 1–14. In the second cycle capecitabine was given from day 3 to 14, the first 2 days of capecitabine being omitted to allow pharmacokinetics samples to be taken. Subsequent cycles combined capecitabine twice daily (b.i.d.) from day 1 to 14 with docetaxel given on day 1. The prophylactic use of growth-factors was not allowed.

### Routine comedication

Oral dexamethasone (8 mg) or methylprednisolone (32 mg) was given to all patients 12 and 3 h before docetaxel infusion, and then 12 and 24 h after the end of docetaxel infusion, followed by either 8 mg or 32 mg twice daily for an additional 3 days. No standard i.v. anti-emetic prophylaxis was given.

### Pharmacokinetic studies

For pharmacokinetic analyses, blood samples (5 ml) were obtained from an indwelling i.v. canula in the contralateral arm, and collected in haemogard vacutainer tubes (Becton Dickinson, Meylan, France) containing EDTA as an anticoagulant. On days 1 and 14 blood was taken to measure levels of capecitabine and its metabolites, and on days 1–3 and 22–24 to measure docetaxel levels. Blood was collected on days 1–3 to explore any possible interaction between the two drugs. Blood samples (5 ml) were taken before the morning capecitabine dose and at 0.5, 1, 2, 3, 4, 5 and 7 h after drug administration; a final blood sample was taken at 10 h after the morning capecitabine dose, but prior to the evening drug administration. Blood samples (5 ml) for docetaxel pharmacokinetics were taken before administration of docetaxel, halfway through the infusion (0.5 h) and within 5 min of completing the infusion (at 1 h). Additional samples were taken at 1.5, 2, 3, 5, 7, 10, 24, 30 and 48 h after the start of infusion. On day 14 blood was collected as described above, for the determination of capecitabine pharmacokinetic parameters without potential interference from docetaxel. On days 22–24 blood was collected as described above, for the determination of docetaxel pharmacokinetic parameters without potential interference from capecitabine.

Concentrations of capecitabine and its metabolites 5′-DFCR, 5′-DFUR, 5-FU and α-fluoro-β-alanine (FBAL) in plasma were determined by liquid chromatography with mass-spectrometic

*© 2000 Cancer Research Campaign* 

detection (LC-MS) as described previously (Reigner et al, 1999).

Pharmacokinetics of capecitabine and its metabolites were estimated by model-independent analysis using SAS Companion for the Microsoft Environment version 6 (SAS Institute Inc., Cary, NC, USA). The area under the plasma concentration–time curve (AUC) was estimated by the trapezoidal rule using data until the last measurable concentration, and was extrapolated to infinity using the ratio of drug level at the last sampling point and the apparent rate constant of the terminal phase. The terminal elimination half-life of the compounds was calculated using least-squares linear regression of the final part of the plasma concentration–time plot. Peak plasma concentrations ($C_{max}$) and the time to reach the peak concentration ($t_{max}$) were also determined graphically.

Plasma samples for docetaxel analysis were prepared by a single solvent extraction and assayed by a validated reversed-phase high-performance liquid chromatographic (HPLC) method with UV detection as reported elsewhere (Loos et al, 1997). Docetaxel concentration–time curves were analysed by determination of slopes and intercepts of plotted curves with multi-exponential functions. Initial parameter estimates were determined by the SIPHAR version 4.0 program (Simed, Creteil, France) and improved using an iterative numerical algorithm based on Powell's method. Model discrimination was assessed by a variety of considerations, including visual inspection of the predicted curves, dispersion of residuals, minimization of the sum of weighted squares residuals, and the Akaike and Schwartz information criteria (Rowland and Tozer, 1995). In all cases, concentration–time profiles were best fitted to a bi-exponential model after zero-order input with weighting according to $l/Y_{obs}$. Final values of the iterated parameters of the best fit equation were used to calculate kinetic parameters using standard equations (Rowland and Tozer, 1995).

### Statistical analysis

Kinetic parameters for capecitabine, its metabolites and docetaxel are reported as arithmetic mean values ± standard deviation or as median values ($t_{max}$ only). Variability in parameters between the various docetaxel dose levels was evaluated using the Kruskal–Wallis statistic followed by a Dunn's test. Interpatient differences in pharmacokinetics were assessed from the coefficient of variation (CV), expressed as the ratio of the standard deviation and the observed mean. To test parameter differences for statistical significance among treatment courses, a two-tailed paired Student's *t*-test was performed. Probability values of less than 0.05 were regarded as statistically significant. All statistical calculations were performed using NCSS (version 5.X; Dr Jerry Hintze, Kayesville, UT, USA) and STATGRAPHICS Plus (version 2; Manugistics Inc., Rockville, MA).

### Doses

In this study dose escalation was performed in two phases, firstly combining a fixed dose of capecitabine with increasing doses of docetaxel. In the second phase the dose of capecitabine was increased with a fixed dose of docetaxel demonstrated in the first phase of escalation to be tolerable. The following dose levels of capecitabine/docetaxel were explored: 825 mg m$^{-2}$ b.i.d.$^{-1}$ 75 mg m$^{-2}$; 825 mg m$^{-2}$ b.i.d 85 mg m$^{-2}$; 825 mg m$^{-2}$ b.i.d. 100 mg m$^{-2}$; 1000 mg m$^{-2}$ b.i.d. 100 mg m$^{-2}$; 1000 mg m$^{-2}$ b.i.d. 75 mg m$^{-2}$; 1250 mg m$^{-2}$ b.i.d. 75 mg m$^{-2}$.

The docetaxel and capecitabine doses were escalated according to a pre-established schedule. Dose escalation was continued until DLTs were experienced in the first 2 cycles of treatment in two or more of six patients, which was defined as the MTD. DLT was defined as: (1) granulocytes $< 0.5 \times 10^9$ l$^{-1}$ for more than 7 days; (2) grade 4 granulocytopenia with complications such as fever or other non-haematological toxicities; (3) gastrointestinal toxicity > grade 2; (4) skin toxicity (i.e. hand–foot syndrome) > grade 2.

### RESULTS

A total of 33 patients entered this study. Patient characteristics are given in Table 1. The most frequent tumour types were colorectal cancer, adenocarcinoma of unknown primary (ACUP) and breast cancer. All patients were evaluable for toxicity and tumour response. Eight patients were not evaluable for pharmacokinetics; in six patients blood was collected only on days 1–3, while in two patients samples at essential time points were missing. The dose levels studied, the number of patients at each dose level and the number of evaluable courses at each dose level are given in Table 2.

A total of 123 courses were assessable for toxicity. No DLTs were reported for the first 2 cycles at dose levels I and II. Significant toxicities were observed in cycle 1 at dose levels III and IV, consisting of febrile neutropenia. In one patient severe anorexia was reported in cycle 1 at dose level IV, and another patient showed in cycle 1 of dose level VI a reversible hepatotoxicity grade 3 consisting of an elevation of the serum bilirubin level.

**Table 1** Patient characteristics

| | |
|---|---|
| Patients treated | 33 |
| Age | |
| Median (range) | 57 (33–74) |
| Karnofsky PS | |
| Median (range) | 80 (70–100) |
| Sex | |
| Male/female | 15/18 |
| Previous chemotherapy | |
| None | 13 |
| 1 regimen | 18 |
| 2 regimens | 2 |
| Tumour type | |
| Colorectal | 9 |
| ACUP | 6 |
| Breast | 3 |
| Miscellaneous | 15 |

**Table 2** Patient accrual

| Dose level | Capecitabine (mg m$^{-2}$ b.i.d.) | Docetaxel (mg m$^{-2}$) | No. patients | No. cycles | Range |
|---|---|---|---|---|---|
| I | 825 | 75 | 4 | 14 | 1–6 |
| II | 825 | 85 | 6 | 32 | 3–6 |
| III | 825 | 100 | 6 | 16 | 1–6 |
| IV | 1000 | 100 | 5 | 14 | 1–6 |
| V | 1000 | 75 | 6 | 24 | 2–6 |
| VI | 1250 | 75 | 6 | 23 | 1–6 |
| Total | | | 33 | 123 | |

© 2000 Cancer Research Campaign

MTD was reached when the capecitabine dose was increased to 1000 mg $m^{-2}$ b.i.d. (dose level IV). At this dose level all patients showed grade 2–3 asthenia that was considered dose limiting. The docetaxel dose was then reduced to 75 mg $m^{-2}$, while the capecitabine dose was escalated to full single-agent dose (dose level VI). DLTs as defined in the protocol were not encountered. No toxic deaths were reported.

### Haematological toxicity

The relevant haematological toxicities are shown in Table 3. Neutropenia grade 3 and 4, lasting < 7 days were observed at all dose levels in 68% (range 31–88%) of all courses, but febrile neutropenia requiring hospital admission was reported in only three courses (2.4%). Anaemia grades 1 and 2 were common at all dose levels and occurred in 89% of all courses; more severe anaemia was not reported. Thrombocytopenia grade 4 requiring platelet transfusion was only observed in 1 course.

### Non-haematological toxicity

The most common non-haematological toxicities are shown in Table 4. Nausea and vomiting were usually mild (grade 1 and 2) and occurred in 33% and 11% of courses respectively. Grade 3 nausea was observed in three courses at dose levels II, III and VI, while vomiting grade 3 was only reported in one course at dose level VI. Mucositis grade 1–2 was documented in 42% of courses. Severe mucositis was observed in two courses at dose levels II and IV respectively. Diarrhoea grade 1–2 was reported in 33% of courses and was severe in one course at dose level V. Asthenia (or fatigue) was an important side-effect; grade 2–3 asthenia was documented in 26% of courses. At dose level IV grade 2–3 asthenia was observed in all patients in 93% of courses, which was considered dose-limiting. Alopecia was common at all dose levels. Hand–foot syndrome was reported in 26.8% of courses which required dose reduction in three patients and treatment delay in two patients. Nail toxicity was observed in 24% of courses and

**Table 3** Haematological toxicity[a]

| Dose level | | I | II | III | IV | V | VI | Total (%) |
|---|---|---|---|---|---|---|---|---|
| Capecitabine (mg $m^{-2}$ b.i.d.) | | 825 | 825 | 825 | 1000 | 1000 | 1250 | |
| Docetaxel (mg $m^{-2}$) | | 75 | 85 | 100 | 100 | 75 | 75 | |
| No. evaluable courses | | 14 | 32 | 16 | 14 | 24 | 23 | 123 |
| Neutropenia | G3 | 3 | 13 | 1 | 2 | 6 | 4 | 29 (24) |
| | G4 | 6 | 15 | 4 | 9 | 7 | 13 | 54 (44) |
| Febrile neutropenia | | – | 1 | 1 | 1 | – | – | 3 (2.4) |
| Thrombopenia | G1–2 | – | 4 | – | – | – | 1 | 5 (4) |
| | G3–4 | – | 1 | – | – | – | – | 1 (0.8) |
| Anaemia G1–2 | | 13 | 28 | 11 | 13 | 23 | 22 | 110 (89) |

[a]No. of courses affected/total courses.

**Table 4** Non-haematological toxicity[a]

| Dose level | | I | II | III | IV | V | VI | Total (%) |
|---|---|---|---|---|---|---|---|---|
| Capecitabine (mg $m^{-2}$ b.i.d.) | | 825 | 825 | 825 | 1000 | 1000 | 1250 | |
| Docetaxel (mg $m^{-2}$) | | 75 | 85 | 100 | 100 | 75 | 75 | |
| No. evaluable courses | | 14 | 32 | 16 | 14 | 24 | 23 | 123 |
| Nausea | G 1–2 | 3 | 15 | 4 | 7 | 8 | 3 | 40 (33) |
| | G 3–4 | – | 1 | 1 | – | – | 1 | 3 (2.4) |
| Vomiting | G 1–2 | 2 | – | 2 | 2 | 5 | 3 | 13 (11) |
| | G 3–4 | – | – | – | – | – | 1 | 1 (0.8) |
| Diarrhoea | G 1–2 | 8 | 9 | 4 | 4 | 13 | 3 | 41 (33) |
| | G 3–4 | – | – | – | – | 1 | – | 1 (0.8) |
| Mucositis | G 1–2 | 6 | 11 | 8 | 8 | 13 | 5 | 51 (42) |
| | G 3–4 | – | 1 | – | 1 | – | – | 2 (1.6) |
| Asthenia | G 1 | 5 | 11 | 4 | – | 3 | 7 | 30 (24) |
| | G 2–3 | 2 | 4 | 1 | 13 | 6 | 6 | 32 (26) |
| Alopecia | G 1–2 | 9 | 24 | 12 | 9 | 18 | 18 | 90 (73) |
| Hand–foot | G 1–2 | 4 | 15 | 3 | – | 8 | 2 | 32 (26) |
| | G 3–4 | – | 1 | – | – | – | – | 1 (0.8) |
| Nail toxicity[b] | | 4 | 7 | 1 | 4 | 6 | 7 | 29 (24) |
| Neurotoxicity | G 1–2 | 4 | 7 | 5 | 4 | 5 | 1 | 26 (21) |
| Oedema | G 1–2 | 6 | 2 | – | – | 1 | 1 | 10 (8) |
| Allergy | | – | 3 | 3 | 1 | 1 | 1 | 9 (7) |

[a]No. of courses affected/total courses; [b]see text.

*© 2000 Cancer Research Campaign*

was complicated with paronychia in four patients. One patient treated at dose level II developed septic paronychia that required dose reduction. Docetaxel related toxicities like neuropathy, oedema and allergy were mild and never a reason to stop therapy.

### Tolerability of multiple cycles

Three patients at dose level II, one patient at dose level IV and one patient at dose level V underwent dose reduction. The method of dose reduction was not pre-established. In two of the three patients at dose level II the dose of both drugs was reduced by 25% because of hand–foot syndrome in cycle 3 in one patient and in cycle 4 in the other patient. One patient at dose level II developed thrombocytopenia grade 4 in cycle 4 that required a dose reduction by 50% of only capecitabine, because it was assumed that capecitabine rather than docetaxel contributed to the occurrence of thrombo-cytopenia. The patient at dose level IV underwent a dose reduction by 25% of both drugs in cycle 2 because of grade 2 nausea and anorexia. The patient at dose level V underwent a dose reduction by 25% of both drugs because of hand–foot syndrome in cycle 4. Treatment was delayed because of hand–foot syndrome in two patients at dose level II after cycle 3 and 4 respectively. Both hand–foot syndrome and nail toxicity were sometimes problematic with prolonged treatment. However, no other cumulative toxicity was observed. The dose intensity of this treatment schedule was high at all dose levels (0.95–1), but lower at dose level IV (0.84).

### Pharmacokinetics

In the pharmacokinetic calculations only patients with complete AUC were taken into consideration. Capecitabine pharmacokinetics were characterized by a rapid absorption after oral dosing, with peak plasma levels occurring at approximately 1 h. In the majority of patients the main circulating compounds were 5′-DFUR (the immediate precursor of 5-FU) and the 5-FU metabolite FBAL. The pharmacokinetic characteristics of capecitabine and the metabolities are listed in Table 5 as a function of the dose. In general, the pharmacokinetics demonstrated high interpatient variability. Overall the kinetic data of capecitabine and the metabolites in the presence of docetaxel indicate a very minor effect of co-treatment with the taxane (Table 5). However, whereas identical parameters were observed for capecitabine, 5′-DFCR, 5′-DFUR and FBAL between study courses, the systemic exposure to 5-FU tended to decrease in the presence of docetaxel. This effect was particularly striking at the 1250 mg $m^{-2}$ b.i.d. dose level, resulting in a 1.8- and 1.9-fold lower values for $C_{max}$ and AUC (Table 5).

The plasma concentration-time profiles for docetaxel were similar with and without capecitabine co-treatment. In both cases, disposition phases of docetaxel exhibited a bi-exponential decay and could be best fitted to a two-compartmental model. The mean estimated pharmacokinetic parameters of docetaxel for both study courses are summarized as a function of the treatment cohort in Table 6.

Substantial interpatient kinetic variability was apparent with values for the coefficient of variation up to 50%. There were no significant differences in dose-normalized pharmacokinetic parameters between the docetaxel dose levels, as shown by the dose-independent values for docetaxel plasma clearance. The mean overall total body clearance of docetaxel across all dose levels without capecitabine co-treatment was 25.4 ± 8.79 l $h^{-1}$ $m^{-2}$ (mean ± standard deviation). Docetaxel pharmacokinetics were not significantly altered by co-treatment with capecitabin ($P < 0.05$ for all kinetic parameters using two-tailed Student's *t*-test), indicating no mutual kinetic interaction between these two drugs (Table 6).

### Responses

Two complete responses were documented in a patient with ACUP (total disappearance of previously found metastatic sites: lymph-nodes, adrenal glands and soft tissue) and in another patient with gastric cancer (metastatic sites: lymph nodes and pancreatic region). Time to progression was 8 months in both patients. Partial

**Table 5** Summary of paired capecitabine and metabolities pharmacokinetics in the presence or absence of docetaxel[a]

| **Capecitabine (mg $m^{-2}$ b.i.d.)**<br>**Docetaxel (mg $m^{-2}$)** | **825**<br>**75,100** | **825**<br>**–** | **1000**<br>**75,100** | **1000**<br>**–** | **1250**<br>**75** | **1250**<br>**–** |
|---|---|---|---|---|---|---|
| Capecitabine | | | | | | |
| $t_{1/2}$ (h) | 0.59 ± 0.31 | 0.55 ± 0.31 | 0.69 ± 0.29 | 0.70 ± 0.32 | 0.58 ± 0.28 | 0.66 ± 0.38 |
| AUC (µg h $ml^{-1}$) | 5.19 ± 3.01 | 3.82 ± 1.31 | 5.58 ± 2.96 | 5.66 ± 2.38 | 7.01 ± 4.21 | 6.39 ± 0.83 |
| 5′-DFCR | | | | | | |
| $t_{1/2}$ (h) | 0.82 ± 0.24 | 0.86 ± 0.38 | 0.80 ± 0.13 | 0.87 ± 0.20 | 0.87 ± 0.24 | 0.76 ± 0.09 |
| AUC (µg h $ml^{-1}$) | 3.56 ± 2.35 | 4.35 ± 2.65 | 7.61 ± 3.35 | 8.23 ± 3.95 | 11.9 ± 5.24 | 9.78 ± 1.08 |
| 5′-DFUR | | | | | | |
| $t_{1/2}$ (h) | 0.65 ± 0.12 | 0.70 ± 0.23 | 0.79 ± 0.32 | 0.71 ± 0.18 | 0.81 ± 0.27 | 0.63 ± 0.12 |
| AUC (µg h $ml^{-1}$) | 12.3 ± 3.20 | 10.8 ± 3.24 | 12.6 ± 2.27 | 11.8 ± 3.78 | 16.1 ± 3.70 | 16.9 ± 2.37 |
| 5-FU | | | | | | |
| $t_{1/2}$ (h) | 0.71 ± 0.16 | 0.70 ± 0.17 | 0.87 ± 0.47 | 0.72 ± 0.18 | 0.92 ± 0.50 | 0.68 ± 0.09 |
| AUC (µg h $ml^{-1}$) | 0.36 ± 0.19 | 0.41 ± 0.15 | 0.48 ± 0.15 | 0.61 ± 0.31 | 0.42 ± 0.13 | 0.80 ± 0.28 |
| FBAL | | | | | | |
| $t_{1/2}$ (h) | 2.43 ± 0.36 | 2.83 ± 0.45 | 2.76 ± 0.61 | 2.96 ± 0.50 | 2.11 ± 0.36 | 2.24 ± 0.11 |
| AUC (µg h $ml^{-1}$) | 16.8 ± 5.38 | 21.1 ± 4.85 | 23.3 ± 9.55 | 25.4 ± 9.65 | 20.8 ± 2.29 | 20.4 ± 0.61 |

[a]Data were obtained from 16, ten and four patients treated at capecitabine dose levels of 825, 1000 and 1250 mg $m^{-2}$ b.i.d. respectively. Kinetic terms are arithmetic mean values ± standard deviation ($t_{1/2}$, and AUC). Abbreviations: $t_{1/2}$, terminal elimination half-life; AUC, area under the plasma concentration–time curve; 5′-DFCR, 5′-deoxy-5-fluorocytidine; 5′-DFUR, 5′-deoxy-5-fluorouridine; 5-FU, 5-fluorouracil; ($FUH_2$), dihydro-5-fluorouracil; FBAL, α-fluoro-β-alanine

 © 2000 Cancer Research Campaign

**Table 6** Summary of paired docetaxel pharmacokinetics in the presence or absence of capecitabine[a]

| Docetaxel (mg m$^{-2}$) | Capecitabine (mg m$^{-2}$ b.i.d.) | *n* | $C_{max}$ (μg ml$^{-1}$) | $t_{1/2}$ (h) | AUC (μg h ml$^{-1}$) | CL (l h$^{-1}$ m$^{-2}$) | $V_{ss}$ (l m$^{-2}$) (h) |
|---|---|---|---|---|---|---|---|
| 75 | 825 | 3 | 2.25 ± 0.23 | 2.83 ± 1.19 | 2.47 ± 0.35 | 30.9 ± 4.53 | 41.3 ± 12.9 |
| 75 | – | 3 | 2.10 ± 1.04 | 1.81 ± 0.96 | 2.32 ± 1.16 | 32.8 ± 15.6 | 33.3 ± 15.9 |
| 85 | 825 | 4 | 2.57 ± 0.53 | 7.52 ± 5.42 | 3.74 ± 1.09 | 25.0 ± 7.92 | 63.1 ± 24.4 |
| 85 | – | 4 | 2.64 ± 0.47 | 5.88 ± 2.87 | 3.37 ± 0.97 | 27.7 ± 8.86 | 68.5 ± 24.8 |
| 100 | 825 | 4 | 3.28 ± 0.78 | 12.2 ± 9.24 | 4.45 ± 1.26 | 24.5 ± 7.00 | 123 ± 89.9 |
| 100 | – | 4 | 3.96 ± 1.76 | 9.81 ± 4.62 | 6.07 ± 2.48 | 18.7 ± 5.44 | 75.9 ± 44.3 |
| 100 | 1000 | 3 | 3.78 ± 0.56 | 12.4 ± 9.24 | 5.09 ± 0.97 | 20.4 ± 3.86 | 86.7 ± 1.75 |
| 100 | – | 3 | 3.73 ± 0.06 | 9.39 ± 2.88 | 5.13 ± 0.51 | 19.7 ± 2.01 | 72.0 ± 28.5 |
| 75 | 1000 | 6 | 2.29 ± 0.11 | 6.90 ± 3.71 | 3.05 ± 0.56 | 25.4 ± 4.44 | 82.0 ± 42.8 |
| 75 | – | 6 | 2.54 ± 0.70 | 5.40 ± 2.41 | 3.45 ± 1.29 | 24.3 ± 7.11 | 57.4 ± 14.2 |
| 75 | 1250 | 5 | 2.34 ± 0.67 | 8.15 ± 9.05 | 3.25 ± 1.08 | 26.1 ± 9.45 | 95.9 ± 100 |
| 75 | – | 5 | 2.78 ± 0.55 | 8.46 ± 9.36 | 3.16 ± 0.70 | 25.1 ± 6.23 | 115 ± 134 |

[a]Kinetic terms are mean values ± standard deviation. Abbreviations: *n*, number of patients with complete paired kinetic data; $C_{max}$, peak plasma level; $t_{1/2}$, terminal elimination half-life; AUC, area under the plasma concentration-time curve; CL, total body clearance; $V_{ss}$, volume of distribution at steady state.

responses were reported in two patients with breast cancer (metastatic sites: liver in 1 patient and skin and lymphnodes in the other patient) and in one patient with colon cancer (metastatic sites: liver and peritoneal). Time to progression in these patients was 6 months for the patients with breast cancer and 9.5 months for the patient with colon cancer.

### DISCUSSION

Capecitabine is a new orally available tumour-selective fluoropyrimidine carbamate, that is bioactivated by a three-enzyme process to provide prolonged high levels of the active moiety, 5-FU, in tumour cells (Investigational drug brochure: capecitabine 1997). Capecitabine is active against advanced breast cancer that is resistant to anthracyclines and taxanes (Blum et al, 1999). In most cases, however, combination therapy is prefered to single-agent treatment. Docetaxel was selected for the combination with capecitabine, since it is probably the most active single agent in the treatment of breast cancer (Ten Bokkel Huinink et al, 1994; Chevallier et al, 1995). In addition, docetaxel and capecitabine have toxicity profiles that only partially overlap. In this phase I study we have shown that capecitabine and docetaxel can be combined safely and effectively, giving both agents at doses where they possess single-agent activity.

Dose escalation was performed in 2 phases, firstly combining a fixed dose of capecitabine with increasing doses of docetaxel. In the second phase the dose of capecitabine was increased with a fixed dose of docetaxel demonstrated to be tolerable in the first phase of dose escalation. A starting dose of 75 mg m$^{-2}$ of docetaxel was chosen as phase I studies showed this dose to be active with a favourable toxicity profile (Pronk et al, 1995). A dose of 825 mg m$^{-2}$ bid of capecitabine when given as an intermittent schedule was well tolerated and active in phase I studies (Budman et al, 1998; Mackean et al, 1998). These starting doses were combined as it was anticipated that this combination would be active and tolerable.

The most important non-haematological toxicity was asthenia which was considered dose-limiting when 1000 mg m$^{-2}$ b.i.d. of capecitabine was combined with 100 mg m$^{-2}$ of docetaxel (dose level IV). Other DLTs as foreseen in the protocol were not encountered. The major haematological toxicity of the combination was neutropenia grade 3 and 4, lasting < 7 days, which occurred in 68% of all courses. However, in only three courses was neutropenia complicated with fever requiring hospital admission. Gastrointestinal toxicity was frequent but again usually mild. Hand–foot syndrome, which is characteristic of capecitabine, was reported in 26.8% of courses; in most cases this was not severe and only required dose reduction in three patients and treatment delay in two patients. The incidence of docetaxel-specific toxicities like fluid retention and allergy was low and did not constitute a major clinical problem, probably because all patients received corticosteroid comedication. Neurotoxicity was also mild and only occurred in 21% of courses, which is less than reported in patients treated with docetaxel as a single agent (Hilkens et al, 1996). However, nail toxicity was sometimes problematic in patients with prolonged treatment.

The evaluation of pharmacokinetic interaction when combining novel, active chemotherapy agents is extremely relevant. In this study the possible up-regulation of TP by taxanes raised the possibility that exposure to 5-FU may be increased by co-administration of docetaxel. Pharmacokinetic studies were performed for capecitabine as a single agent and in the presence of docetaxel. Plasma peak concentrations for the drug were reached shortly after oral dosing. As predicted by earlier investigations, capecitabine was extensively metabolized by hepatic carboxylesterases into 5′-DFCR with subsequent cytidine deamination to form the 5-FU precursor 5′-DFUR (Budman et al, 1998; Mackean et al, 1998). The latter compound was, together with the 5-FU metabolite FBAL, the main circulating compound in the majority of patients. The pharmacokinetics of capecitabine showed high interpatient variability and were highly consistent with recently published values obtained in patients treated at a single-agent dose of 1657 mg m$^{-2}$ day$^{-1}$ (Budman et al, 1998). Overall, the kinetic data of capecitabine and its metabolites were similar for capecitabine as a single agent and in the presence of docetaxel. However, the systemic exposure to 5-FU tended to decrease in the presence of docetaxel. This effect was particularly striking at the 1250 mg m$^{-2}$ b.i.d. dose level. More pharmacokinetic studies will be needed to explain the significance of this observation.

The plasma concentration-time profiles for docetaxel given as a single agent and with capecitabine were similar. The interpatient kinetic variability, particularly at the 75 mg m$^{-2}$ dose level, was

© 2000 Cancer Research Campaign 

substantial which could in part be accounted for by missing samples at essential time points in some patients. (There was no systemic reason for the missing data.) The mean overall total body clearance of docetaxel as a single agent across all dose levels was 25.4 ± 8.79 l $h^{-1}$ $m^{-2}$ (mean ± standard deviation). This is consistent with previously published values obtained in phase 1 clinical trials on docetaxel as a single agent (Bruno and Sanderink 1993). These data indicate that capecitabine has no significant effect on docetaxel pharmacokinetics.

The combination of capecitabine and docetaxel is clearly active with two complete responses (one patient with ACUP, one with gastric cancer) and three partial responses (two with breast cancer, one with colon cancer). The antitumour activity of capecitabine as a single agent in patients with advanced breast cancer has been demonstrated in series of phase II trials. A randomized phase II study in women aged 55 years or older compared capecitabine with CMF as first-line treatment (O'Shaugnessy et al, 1998); preliminary results showed that capecitabine monotherapy is at least comparable with CMF combination chemotherapy. However, severe hand–foot syndrome and diarrhoea were more frequent in the capecitabine treatment arm. In a multicenter phase II trial in patients with paclitaxel-refractory metastatic breast cancer, who were all also pretreated with anthracyclines, patients received a dose of 2510 mg $m^{-2}$ $day^{-1}$ given for 2 weeks followed by a 1-week rest period, repeated every 3 weeks (Blum et al, 1998). The toxicity profile was acceptable and the response rate was 20%, with a median response duration of 8.1 months and a median survival of 12.8 months. The median time to disease progression was 93 days. This study shows that capecitabine is active in paclitaxel/anthracycline resistant breast cancer, and suggests that there is no cross-resistance between capecitabine and taxanes, a further justification for the use of capecitabine and docetaxel in combination. The combination of capecitabine with paclitaxel is also under investigation in a phase I study in patients with previously treated metastatic breast cancer (Khoury et al, 1998). The combination appears to be active, even in patients who had prior bone marrow transplantation.

Based on the experience obtained in this phase I study, repeated cycles of capecitabine 825 mg $m^{-2}$ bid combined with docetaxel 100 mg $m^{-2}$ or capecitabine 1250 mg $m^{-2}$ b.i.d. with docetaxel 75 mg $m^{-2}$ are both feasible. A randomized phase III study comparing the combination of capecitabine 1250 mg $m^{-2}$ and docetaxel 75 mg $m^{-2}$ with docetaxel 100 mg $m^{-2}$ as a single agent is ongoing in patients with metastatic breast cancer as first-line treatment.

### REFERENCES

Aamdal S, Wolff I, Kaplan S, Paridaens R, Kerger R, Schachter J, Wanders J, Franklin H and Verweij J (1994) Taxotere in advanced malignant melanoma: a phase II trial of the EORTC Clinical Trials Group. *Eur J Cancer* **30A**: 1061–1064

Aapro MS, Zulian G, Alberto P, Bruno R, Oulid-Aissa D and Le Bail N (1992) Phase I and pharmacokinetic study of RP 56976 in a new ethanol-free formulation of Taxotere. *Ann Oncol* **3 (suppl 5)**: 208

Bisset D, Setanoians A, Cassidy J, Graham MA, Chadwick GA, Wilson P, Auzannet V, Le Bail N, Kaye SB and Kerr DJ (1993) Phase I and pharmacokinetic study of Taxotere (RP 56976) administered as a 24-hour infusion. *Cancer Res* **53**: 523–527

Blum JL, Jones SE, Buzdar AU, LoRusso PM, Kuter I, Vogel C, Osterwalder B, Burger HU, Brown C and Griffin T (1999) Multicenter phase II study of capecitabine in paclitaxel-refractory metastatic breast cancer. *J Clin Oncol* **17**: 485–493

Ten Bokkel-Huinink WW, Prove AM, Piccart M, Steward W, Tursz T, Wanders J, Franklin H, Clavel M, Verweij J, Alaki M, Bayssas M and Kaye SB (1994) A phase II trial with docetaxel (Taxotere) in second-line treatment with chemotherapy for advanced breast cancer. A study of the EORTC Early Clinical Trials Group. *Ann Oncol* **5**: 527–532

Brundage MD, Pater JL and Zee B (1993) Assessing the reliability of two toxicity scales: implications for interpretating toxicity data. *J Natl Cancer Inst* **85**: 1138–1148

Bruno R and Sanderink GJ (1993) Pharmacokinetics and metabolism of Taxotere. In: *Pharmacokinetics and Cancer Chemotherapy*, Workman P and Graham MA (eds), pp. 305–313. Cold Spring Harbor Laboratory: New York, NY

Budman DR, Meropol NJ, Reigner B, Creaven PJ, Lichtman SM, Berghorn E, Behr J, Gordon RJ, Osterwalder B and Griffin T (1998) Preliminary results of a novel oral fluoropyrimidine carbamate. *J Clin Oncol* **16**: 1795–1802

Burris H, Irvin R, Kuhn J, Kalter S, Smith L, Shaffer D, Fields S, Weiss G, Eckardt J, Rodriguez G, Rinaldi D, Wall J, Cook G, Smith S, Vreeland F, Bayssas M, Le Bail N and Von Hoff D (1993) Phase I clinical trial of Taxotere administered as either a 2-hour or 6-hour intravenous infusion every 3 weeks. *J Clin Oncol* **11**: 950–958

Catimel G, Verweij J, Mattijsen V, Hanauske A, Piccart M, Wanders J, Franklin H, Le Bail N, Clavel M and Kaye SB (1994) Docetaxel (Taxotere): an active drug for the treatment of patients with advanced squamous cell carcinoma of the head and neck. *Ann Oncol* **5**: 533–537

Cerny T, Kaplan S, Pavlidis N, Schöffski P, Epelbaum R, Van Meerbeek J, Wanders J, Franklin HR and Kaye S (1994) Docetaxel (Taxotere) is active in non-small cell lung cancer: a phase II trial of the EORTC Clinical Trials Group. *Br J Cancer* **70**: 384–387

Chevallier B, Fumoleau P, Kerbrat P, Dieras V, roche H, Krakowski I, Azli N, Bayssas M, Lentz MA and van Glabbeke M (1995) Docetaxel is a major cytotoxic drug for the treatment of advanced breast cancer: a phase II trial of the Clinical Screening Cooperative Group of the European Organization for Research and Treatment of Cancer. *J Clin Oncol* **13**: 314–322

Extra JM, Rousseau F, Bruno R, Clavel M, Le Bail N and Marty M (1993) Phase I and pharmacokinetic study of Taxotere (RP 56976; NSC 628503) given as a short intravenous infusion. *Cancer Res* **53**: 1037–1042

Findlay M, Van Cutsem E, Kocha W, Allman D, Laffranchi B, Griffin T, Osterwalder B, Dalley D, Pazdur R and Verweij J (1997) A randomized phase II study of Xeloda (Capecitabine) in patients with advanced colorectal cancer. *Proc Am Soc Clin Oncol* **16**: 227a (abstract 798)

De Forni M, Rougier P, Adenis A, Ducreux M, Djazouli K, Adams D, Clouet P, Blanc C, Bayssas M, Bonneterre J and Armand JP (1994) Phase II study of Taxotere in locally advanced and/or metastatic pancreatic cancer. *Ann Oncol* **5 (suppl 5)**: 509

Fossella FV, Lee JS, Shin DM, Calayag M, Huber M, Perez-Soler R, Murphy WK, Lippman S, Benner S, Glisson B, Chasen M, Ki Hong W and Raber M (1995) Phase II study of docetaxel for advanced or metastatic platinum-refractory non-small cell lung cancer. *J Clin Oncol* **13**: 645–651

Gueritte-Voegelein F, Guenard D, Lavelle F, Le Goff MT, Mangatal L and Poitier P (1991) Relationships between the structure of Taxol analogues and their antimitotic activity. *J Med Chem* **34**: 992–998

Hilkens PHE, Verweij J, Stoter G, Vecht Ch, Van Putten WLJ and van den Bent MJ (1996) Peripheral neurotoxicity induced by docetaxel. *Neurology* **46**: 104–108

Investigational drug brochure: Capecitabine (Ro 09-1978) Fourth Version, December 1997

Van Hoesel QGCM, Verweij J, Catimel G, Clavel M, Kerbrat P, van Oosterom AT, Kerger J, Tursz T, van Glabbeke M, Van Pottelsberghe C, Le Bail N, Mouridsen H, for the EORTC Soft Tissue and Bone Sarcoma Group (1994) Phase II study with docetaxel (Taxotere) in advanced soft tissue sarcomas of the adult. *Ann Oncol* **5**: 539–542

Ishikawa T, Sawada N, Sekiguchi F, Fukase Y and Ishitsuka H (1997) Xeloda™ (Capecitabine), a new oral fluoropyrimidine carbamate with an improved efficacy profile over other fluoropyrimidines. *Proc Am Soc Clin Oncol* **16**: 226a (abstract 796)

Khoury P, Villalona-Calero M, Blum J, Diab S, Elledge R, Kraynak M, Moczygemba J, Kromelis P, Morales I, Brown C, Griffin T, Von Hoff D and Rowinsky E (1998) Phase I study of capecitabine in combination with paclitaxel in patients with previously treated metastatic breast cancer. *Proc Am Soc Clin Oncol* **17**: 206a (abstract 793)

Loos WJ, Verweij J, Nooter K, Stoter G and Sparreboom A (1997) Sensitive determination of docetaxel in human plasma by liquid–liquid extraction and reversed-phase high-performance liquid chromatography. *J Chromatogr B* **693**: 437–441

Mackean M, Planting A, Twelves C, Schellens J, Allman D, Osterwalder B, Reigner B, Griffin T, Kaye S and Verweij J (1998) Phase I and pharmacologic study of

© 2000 Cancer Research Campaign intermittent twice-daily oral therapy with capecitabine in patients with advanced and/or metastatic cancer. *J Clin Oncol* **16**: 2977–2985

Miller VA, Rigas JR, Francis PA, Grant SC, Pisters KMW, Venkatraman ES, Woolley K, Heelan RT and Kris MG (1995) Phase II trial of a 75 mg $m^{-2}$ dose of docetaxel with prednisone premedication for patients with advanced non-small-cell lung cancer. *Cancer* **75**: 968–972

O'Shaugnessy J, Moiseyenko V, Bell D, Nabholtz JM, Miles D, Gorbunova V, Laws S, Griffin T and Osterwalder B (1998) A randomized phase II study of Xeloda (Capecitabine) vs CMF as first-line chemotherapy of breast cancer in women > 55 years. *Proc Am Soc Clin Oncol* **17**: 103a (abstract 398)

Pazdur R, Newman RA, Newman BM, Fuentes A, Benvenuto J, Bready B, Moore D, Jaiyesimi I, Vreeland F, Bayssas MMG and Raber MN (1992) Phase I trial of Taxotere: 5-day schedule. *J Natl Cancer Inst* **84**: 1781–1788

Piccart MJ, Klijn J, Paridaens R, Nooij M, Mauriac L, Coleman R, Bontenbal M, Awada A, Selleslags J, Van Vreckem A and Van Glabbeke M (1997) Corticosteroids significantly delay the onset of docetaxel-induced fluid retention: final results of a randomized study of the European Organization for Research and Treatment of Cancer investigational drug branch for breast cancer. *J Clin Oncol* **15**: 3149–3155

Pronk LC, Stoter G and Verweij J (1995) Docetaxel (Taxotere): single agent activity, development of combination treatment and reducing side effects. *Cancer Treatment Rev* **21**: 463–478

Reigner B, Clive S, Cassidy J, Jodrell D, Schulz R, Goggin T, Banken L, Roos B, Utoh M, Mulligan T and Weidekamm E (1999) Influence of the antacid Maalox on the pharmacokinetics of capecitabine in cancer patients. *Cancer Chemother Pharmacol* (in press)

Ringel I and Horwitz SB (1991) Studies with RP 56976 (Taxotere): a semisynthetic analog of Taxol. *J Natl Cancer Inst* **83**: 288–291

Rowinsky EK and Donehower RC (1991) The clinical pharmacology and use of antimicrotubule agents in cancer chemotherapeutics. *Pharmac Ther* **52**: 35–84

Rowland M and Tozer TN (1995) Clinical pharmacokinetics: concepts and applications, 3rd Edition. Media, Williams and Wilkins

Sawada N, Ishikawa T, Fukase Y, Nishida M, Yoshikubo T and Ishitsuka H (1998) Induction of thymidine phosphorylase activity and enhancement of capecitabine efficacy by Taxol/Taxotere in human cancer xenografts. *Clin Cancer Res* **4**: 1013–1019

Schrijvers D, Wanders J, Dirix L, Prove A, Vonck I, Van Oosterom AT and Kaye S (1993) Coping with toxicities of docetaxel (Taxotere). *Ann Oncol* **4**: 610–611

Sulkes A, Smyth J, Sessa C, van Oosterom AT, Vermorken J, Kaye SB, Le Bail N, Verweij J, for the EORTC Early Clinical Studies Group (1994) Docetaxel (Taxotere) in advanced gastric cancer: results of a phase II clinical trial. *Br J Cancer* **70**: 380–383

Taguchi T, Ishitani K, Saitoh K, Kurihara M, Tominaga T, Fukuyama Y, Saigenji K, Toge T, Takashima S, Sugimachi K and Hara Y (1996) A Japanese phase I study of continuous twice daily treatment with capecitabine in patients with advanced and/or metastatic solid tumors. *Ann Oncol* **7**: 87 (abstract 299)

Tomiak E, Piccart MJ, Kerger J, Lips S, Awada A, de Valeriola D, Ravoet C, Lossignol D, Sculier JP, Auzannet V, Le Bail N, Bayssas M and Klastersky J (1993) Phase I study of Taxotere (RP 56976, NSC 628503) administered as a one hour intravenous infusion on a weekly basis. *J Clin Oncol* **12**: 1458–1467

Twelves C, Budman DR, Creaven PJ, Schellens JHM, Kuruma I, Dumont E, Osterwalder B and Reigner BG (1996) Pharmacokinetics (PK) and pharmacodynamics (PD) of capecitabine in two phase I studies. *Proc Am Soc Clin Oncol* **5**: 476 (abstract 1509)

WHO (1979) *Handbook for Reporting Results of Cancer Treatment*. WHO Offset Publication **48**: 22–26

*© 2000 Cancer Research Campaign*
